# Teaching airway teachers: a post-course quantitative and qualitative survey

**DOI:** 10.1186/s12909-023-04912-y

**Published:** 2024-02-07

**Authors:** Irene Steinberg, Sabine Nabecker, Robert Greif, Gerardo Cortese

**Affiliations:** 1 Department of Surgical Sciences, University of Turin, Turin, Italy; 2 Department of Anesthesiology and Pain Management, Sinai Health System, University of Toronto, 600 University Avenue, Toronto, ON M5G 1X5 Canada; 3 School of Medicine, Sigmund Freud University Vienna, Vienna, Austria; 4 Department of Anesthesia, Intensive Care and Emergency, ‘Città Della Salute E Della Scienza’, Turin, Italy

**Keywords:** Airway, Teach the teacher, Survey

## Abstract

**Background:**

Airway management is a crucial skill for many clinicians. Besides mastering the technical skills of establishing a patent airway, human factors including leadership and team collaboration are essential. Teaching these human factors is often challenging for instructors who lack dedicated training. Therefore, the European Airway Management Society (EAMS) developed the Teach-the-Airway-Teacher (TAT) course.

**Methods:**

This online post-course survey of TAT-course participants 2013–2021 investigated the impact of the TAT-course and the status of airway management teaching in Europe. Twenty-eight questions e-mailed to participants (using SurveyMonkey) assessed the courses’ strengths and possible improvements. It covered participants’ and workplace details; after TAT-course considerations; and specifics of local airway teaching. Data were assessed using Excel and R.

**Results:**

Fifty-six percent (119/213) of TAT-participants answered the survey. Most were anaesthetists (84%), working in university level hospitals (76%). Seventy-five percent changed their airway teaching in some way, but 20% changed it entirely. The major identified limitation to airway teaching in their departments was “lack of dedicated resources” (63%), and the most important educational topic was “Teaching non-technical skills” (70%). “Lecturing “ was considered less important (37%). Most surveyed anaesthesia departments lack a standardized airway teaching rotation. Twenty-one percent of TAT-participants rated their departmental level of airway teaching overall as inadequate.

**Conclusions:**

This survey shows that the TAT-course purpose was successfully fulfilled, as most TAT-course participants changed their airway teaching approach and did obtain the EAMS-certificate. The feedback provided will guide future TAT-course improvements to advance and promote a comprehensive approach to teaching airway management.

**Supplementary Information:**

The online version contains supplementary material available at 10.1186/s12909-023-04912-y.

## Background

Being able to secure an airway and to ventilate a patient is a crucial skill for all acute care physicians, especially for anaesthetists, intensive care, and emergency physicians. Being able to maintain oxygenation can mean the difference between life and death for a patient. Airway management is considered a basic competency in acute care medicine but the real experience in airway management may vary greatly between physicians [1].

This is likely due to different factors:

- Only a few sites offer structured airway-teaching rotations. Junior doctors usually acquire basic airway skills and later advanced airway management skills during daily clinical work supervised by busy clinicians [2]. Airway teaching happens “on-the-go”. It is often unstructured and depends usually on available patients’ characteristics rather than following a defined airway management curriculum.
- Airway management has evolved over the past years: Mastering new devices and technical skills to establish a patent airway are still important, but it is only one aspect of teaching airway management. Human factors, especially the ability to coordinate a team, are now considered equally essential and need to be learnt by physicians [3].
- Daily pressures lead to reduced learning opportunities as physicians are pushed to tend to more patients in less time. As a consequence, dedicated airway teachers may encounter difficulties in appropriately maintaining their continuous airway training [4]. In addition, busy clinical environments provide fewer opportunities for airway teaching.
- The training of technical and non-technical airway management skills has been recognized as an important part of healthcare education curricula [5]. However, physicians who deliver airway training programs usually still receive little or no specific training and “teaching the teacher” [4] courses are not widely offered.

Clinical departments should provide airway management curricula that provide the opportunity to learn, train and practice airway management technical skills and human factors [6–8]. Ideally, these curricula follow international guidelines and should include besides didactic theaching of theory the opportunity of simulation based training as well as teaching of the airway skills during daily clinical practice with the ultimate aim to improve patient care and safety.

The ideal formula for maintenance, update and improvement of essential airway management skills throughout a physician’s career is still unclear [4]. In the last decades, efforts have been made to improve teaching, training and providing guidance in airway management. Nowadays, airway management complications are rare, but remain an important contributor to morbidity and mortality of patients if they occur [9]. Poor education and training in airway management might even lead to increased patient morbidity and mortality [10], whereas better training in airway management might improve patient safety [11].

Therefore, the European Airway Management Society (EAMS) developed the Teach-the-Airway-Teacher (TAT) course [12, 13]. Its goals are: to teach participants reliable methods of effective airway teaching, to expand the teachers’ toolbox, and to increase the overall quality of airway management teaching. EAMS TAT-course participants have the chance to discuss and learn new approaches to airway teaching in busy clinical environments [14, 15], by sharing educational principles and training experiences. The TAT-course stimulates collegial exchange between participants [16].

In short, the 22-h TAT-course aims to improve airway management teaching competencies of clinical teachers by providing theoretical background information and following modern didactic principles in a flipped classroom concept. Before the TAT-course, participants are provided short video clips and reading materials that cover adult learning, motivating learners, and the structure of teaching sessions, teaching of complex psychomotor skills and non-technical skills, assessment and feedback, organising and running successful teaching events. The on-site part of the TAT-course discusses and practices in small groups: competencies necessary for a successful airway teacher, how airway management skills and human factors should be taught, providing effective feedback, and features a microteaching session on simulated airway teaching.

During the TAT-course, participants are assessed formatively. The on-site course ends with the development of individual participants’ action plans for their own plans for educational changes and implementation plans at their home institutions. To fulfil the criteria to achieve the EAMS-Airway Teacher certificate, participants are asked to report on the implementation of airway teaching and feedback in a post-TAT-course assignment. This helps increase the transfer of learned competencies into their teaching practice and promotes a long-term effect of the TAT-course.

The first TAT-course took place in Barcelona, Spain in 2013 and since then over 200 clinicians from 35 countries participated successfully. Since 2020, blended learning methods have been introduced in the form of a hybrid TAT-course including an e-learning section to be completed before the TAT-course, and an on-site session consisting of workshops, and specific interactive discussions on airway management-related topics [17]. Continuous feedback is crucial to improve airway management [18] and the TAT-course.

In this study, we aimed to investigate the impact of the TAT-course and the status of airway management teaching in Europe by sending an anonymized post-TAT-course survey to all available TAT-course participants since 2013. This survey aimed to analyse the strengths and weaknesses of the course and to obtain a picture of the current airway management teaching status in Europe.

## Methods

The ethical committee of the University of Torino, Italy reviewed the study and waived its registration because this observational study did not include patients and all data were anonymized (protocol number 00331/2022, 30/06/2022). The study follows the Helsinki Declaration of 1964, which was revised in 2013.

Via the TAT-database of EAMS, all available e-mail addresses of TAT-course participants since the first edition in 2013 were obtained. All valid e-mail addresses were contacted, and TAT-course participants were invited to complete an anonymous online survey on the SurveyMonkey platform (Momentive, California, United States). Informed consent for participation and anonymous data evaluation and publication was obtained via the SurveyMonkey platform before the start of the survey.

The survey was developed by TAT-instructors and consisted of 28 questions (eight closed-answer questions (yes/no/numeric), sixteen multiple choice-questions and four open-answer questions; all questions are displayed in the online Additional file 1 supplement A.

The survey was divided into three sections:

1. TAT-participants and workplace characteristics (two closed-answer questions, five multiple choice-questions).
2. After TAT-course assessment and considerations (three closed-answer questions, seven multiple choice-questions, and two open-answer questions).
3. Specifics of local airway teaching practice and overview (three closed-answer questions, five multiple choice-questions, and two open-answer questions).

The questionnaire was developed in English and was prior to distribution reviewed by six airway management specialists, three Italian, and three German speakers, who provided their feedback. The survey questions were adjusted to assess the face validity of the TAT course as best practice literature has suggested [19].

After the first invitation to complete the survey, three reminders were sent to potential study participants over the following two months. The survey was finally closed after nine weeks. Data was then downloaded from the SurveyMonkey platform.

Closed-answer question results were analysed with the software Excel (Microsoft, Washington, United States), and R (version 4.0, The R Foundation, Vienna, Austria). Continuous data are reported as mean ± standard deviation, and dichotomous or multiple-choice question answers are reported as number (percentage). The qualitative results of open-answer questions were coded by one researcher (SN) and grouped together in common themes.

## Results

### TAT-participants’ characteristics and opinions

We obtained data from 24 TAT-courses, participants originated from 35 countries, 53% were women, 76% completed the post-TAT-course assignment and obtained the EAMS Airway Teacher certificate, and 87% attended an on-site face-to-face TAT-course.

Of the 213 TAT-course participants who had provided e-mail addresses, 119 (56%) answered the survey. Participants had 13 ± 8 years of clinical practice after completing specialist training and had 9 ± 6 years of experience as airway teachers, they did their TAT-course 5 ± 3 years ago. The majority were anaesthetists (84%), who worked in university-level hospitals (76%). Their airway learners were mostly residents (87%), medical students (67%), and specialists (65%). Table 1 displays further details of participants’ and workplaces’ characteristics.

**Table 1:** Participants’ and workplaces’ characteristics. Data are presented as number and percent

	n	%
Working environment		
Anaesthesia	100	84,0
Intensive Care Unit	14	12
Paediatrics	2	2
Research	2	2
Emergency Department	1	1
Type of Hospital		
University level hospital	90	76
Non-University level hospital	28	23
Simulation Centre	1	1
Participants		
Residents	104	87
Medical students	80	68
Specialists	77	66
Certified nurses	49	41
Nursing students	39	33
Paramedics	23	19
Course format		
Face-to-face in presence	104	87
Hybrid blended	15	13

Eighty-four percent of survey participants completed the post-TAT-course assignment and achieved the TAT-Airway teacher certification; 75% reported that they somehow changed their teaching methods after participating in the TAT-course, while 20% changed it completely.

Life-long learning was considered one of the most important duties of an airway teacher (65%), and continuous education was considered a crucial point for career development by 70% of TAT-participants. Further post-TAT-course considerations are displayed in Table 2. The most effective educational topic was “The teaching of non-technical skills” (70%), whereas 37% identified “Lecturing” as the least helpful. Details on the perceived importance of TAT-course topics are displayed in Table 3.

**Table 2:** Post-TAT-course considerations. Data are presented as mean ± standard deviation or number and percent

	n	%
Number of courses taught after TAT	8 (± 9)	
Teaching frequence		
1–2 times/year	42	40
Daily	25	24
Weekly	20	19
Monthly	11	10
Never	7	7
Limitations or challenges as airway teacher after completing the TAT course		
Institution not providing protected teaching time	63	60
Time management	40	38
Missing equipment	39	37
Missing teaching facilities (e.g., rooms)	37	35
Establishing a stimulating learning climate	31	30
Providing effective feedback and debriefing	27	25
Keeping learners attentive and engaged	20	19
Proper assessment	13	12
Interaction with the participants	12	12
Not enough participants	6	6
Effective presentation topics	2	2
Strenghts as a teacher		
My continuous lifelong learning	68	65
Empathy with students	58	55
My airway management skills	57	54
Goal-oriented teaching	53	51
Empathic feedback and debriefing	49	47
Knowledge of educational “theory”	48	46
Engagement with learners	34	32
My status as an airway expert	33	31
Effectiveness in presenting	32	31
Importance of continuous education		
Crucial	74	71
Quite useful	26	25
Sometimes useful	3	3
Unnecessary	1	1
Do not know	1	1

**Table 3:** Helpful and not-so-helpful educational topics during the TAT-course. Data are presented as numbers and percent

	n	%
Most effective educational topic		
Teaching of non-technical skills	74	71
Feedback exercise	65	62
Learning about the role of the teacher	43	41
“Educational Theory”	41	39
Teaching of airway management skills	38	36
Use of blended learning methods	38	36
How to run an airway course	35	33
Micro-teaching exercise	33	31
Lecturing	14	13
Other	2	2
Less helpful educational topic		
Lecturing	39	37
Learning about the role of the teacher	19	18
“Educational Theory”	18	17
How to run an airway course	17	16
Teaching of airway management skills	17	16
Use of blended learning methods	11	11
Micro-teaching exercise	10	10
Other	10	10
Teaching of non-technical skills	8	8
Feedback exercise	6	6

### Airway teaching in the departments

Two-thirds of surveyed departments (68%) organized 1–2 times per year airway management courses, however, 16% never do. We found that 36% of departments offered a dedicated airway teaching rotation for residents, and 16% for specialists. Also, 40% of departments have certified airway teaching clinicians. On-site face-to-face teaching was rated as the best educational modality (75%; compare details in Table 4). Table 5 displays a summary of the qualitative results of the three open-answer questions. The main themes that emerged were changes to adult learning, changes in strategies for improvement, changes in feedback and debriefing, and changes in student-centredness, missing course content, overall recommendations for improvements, next steps after the TAT-course, organisational barriers, lack of teaching personnel, and ideas to overcome barriers.

**Table 4:** Specifics of local airway teaching after the TAT-course (at the participants home institution). Data are number and percent

	n	%
Frequency of departmental course		
1–2 times per year	69	68
Never	16	16
> 2 times per year	16	16
Course modalities availeable		
In presence. Both theory and hands on	44	44
In presence. Hands-on skills	27	27
In presence. Theory	17	17
Blended courses	6	6
Other (no courses organized)	5	5
E-Learning. Skills	2	2
E-learning. Theory	0	0
Favourite teaching modalities		
On-site face to face learning	75	74
Continuous airway programs in my unit	31	31
Clinical rotations with special learning topics	31	31
Airway courses externally	29	29
Dedicated airway fellowships	25	25
Blended hybrid methods	20	20
E-learning, virtual teaching, apps	12	13
Available standardized teaching for residents	36	36
Available standardized refresher for specialists	16	16
Teachers are certified	40	40

**Table 5:** Summary of qualitative results of open-answer questions

What specific changes did you make to your teaching after attending the TAT-course?	What is missing in the TAT-course program?	What is missing to implement at your institution a successful airway-teaching program?
**Adult learning**	**Course content**	**Organisational Barriers**
- Applying learning models - Applying teaching methodology - Planning-ahead of micro-teaching - Small group teaching methods - Formulate precise learning objectives - Encourage learners’ questions - Ensure enough time for practice - Structuring of lessons - Applying of the flipped classroom concept - No more lectures - Know your learners’ level of knowledge	- Focus on improving technical skill teaching - More simulations of teaching situations - Strategies for self-improvement of teachers - More time for feedback - More time for practice - More hands-on practice - More teaching exercises - Introduce new airway techniques - More focus on human factors in crisis situations - Focus on how to train in real situations - Pitfalls to avoid making a course become a disaster - Provide more feedback on teaching performance - Focus on time management during courses - Add oral presentation skills training	- Creation of difficult airway management rotations - Lack of time to attend trainer education - Lack of time to teach - Dedicated time for residents to attend education - Lack of time for organization of structured teaching - Lack of financial support - Lack of technical equipment - Lack of dedicated teaching space (e.g., rooms) - Lack of departmental support for teaching - Lack of support from the surgical partners - Lack of accountability from leadership - Lack of understanding by the hospital leadership - Too much clinical work for airway experts - Lack of support from industry
**Strategies for improvement**		
- Use of positive reinforcement - Focus on non-technical skills teaching - Focus on human factors and patient safety - Introduce Stop and Think Concept - Use of 4-step approach for skills teaching - Emphasize the importance of preparation - Use of simulation scenarios - Use of case discussions - Use of role playing - Providing course material to students - Break-down of complex tasks - Use of cadavers - Help students formulate an action plan - Applying pre- and post-tests - Use of assessments - Enhanced use of media		
	**TAT-course overall**	**Teaching Personnel**
	- Offer more TAT-courses - Spread course out over a longer period - More chances to achieve certification - Provide specific goals of each session - Account for diversity of course participants - Increase duration of whole course - Ensure enough break time - Ensure diverse faculty - Ensure enough time for experience discussions	- Lack of trained trainers - Lack of certified trainers - Lack of interest to teach - Lack of motivation to teach - Lack of know-how - Lack of organisational skills - Lack of continuous education - Lack of clinical airway experts - Lack of assessment
	**Next steps after TAT-course**	**Ideas to overcome barriers**
	- Offer follow-up after certification - Offer refresher TAT-course - Offer advanced TAT-course - Offer TAT-courses for inexperienced specialists - Offer TAT-courses in different languages - Offer TAT-courses in different countries - Offer remote-teaching feedback - Encourage multi-centre expert interaction - Create alumni program	- Creation of an airway laboratory - Use of clinical simulations - Use of short hands-on sessions - Organize theoretical and practical courses - Use e-learning methodology - Use flipped classroom concept - Teach using clinical cases in the hospital - Create courses and rotations for medical students - Create mandatory course to update skills - Use of high-fidelity simulation - Use of cadaver workshops - Use of in-situ simulation - Use of continuous supervision - Invite expert speakers - Rotation of teaching personnel between institutions - Encourage discussions between airway teachers
**Feedback and Debriefing**		
- Use video-recordings of training - Giving effective, structured feedback - Structured Debriefing - Learning Conversation with students - Encourage student interactions		
**Student-Centredness**		
- Adapting teaching to the student’s goals - Communication - Interaction with students - Listening to the problems of students - Offer of further assistance for students - Letting students learn from their mistakes		

### Limitations

**Fig. 1:**
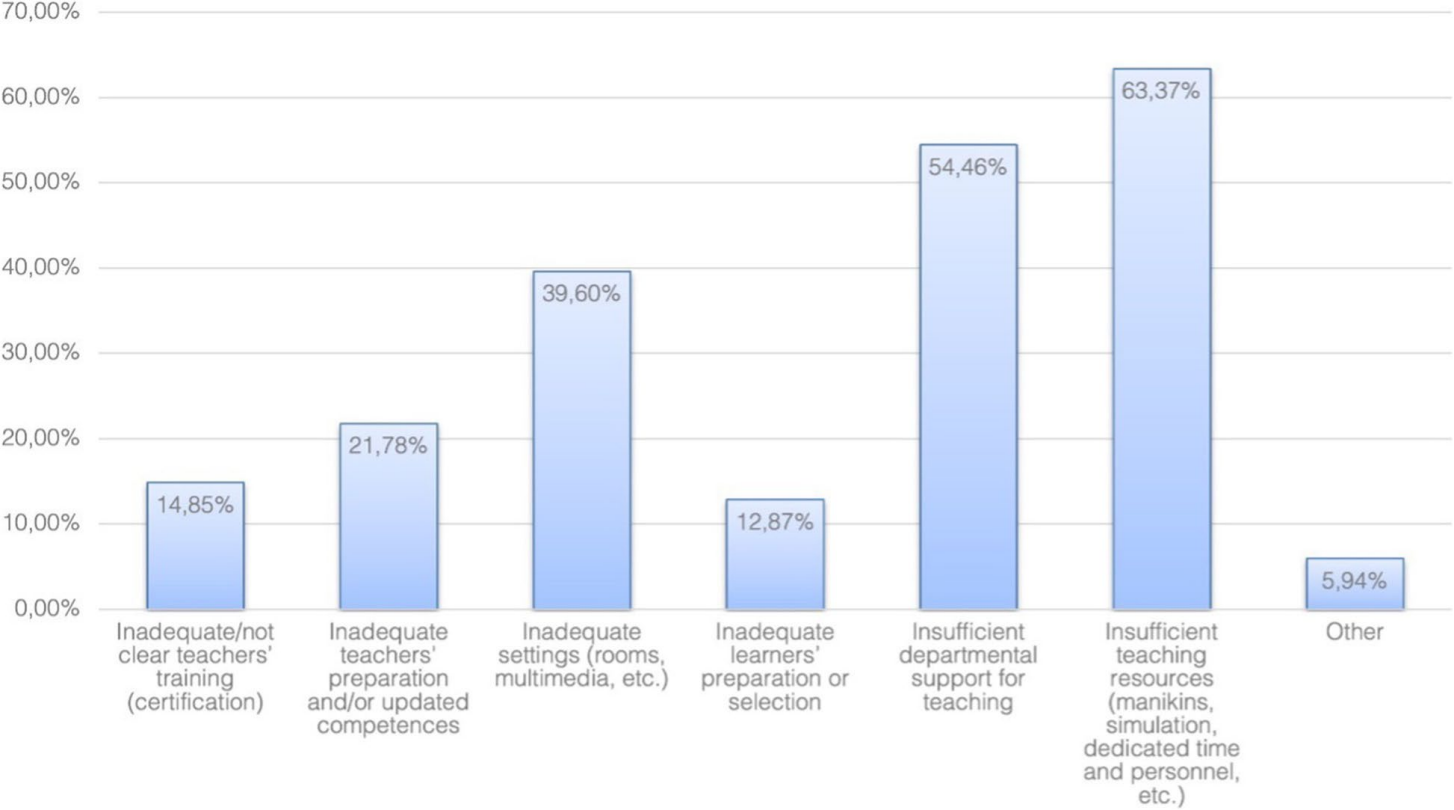
Main limitations to daily airway teaching

**Fig. 2:**
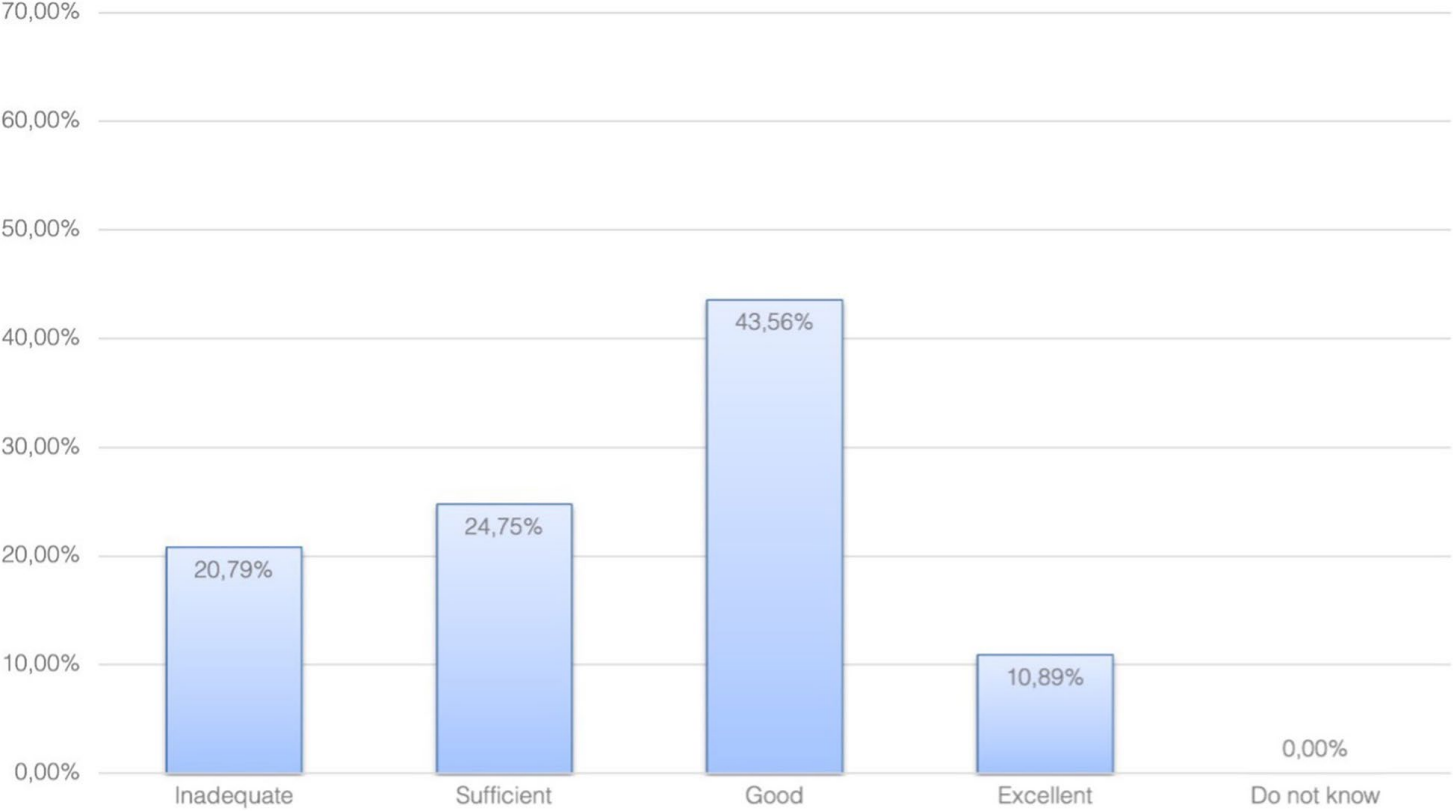
Overall level of preparation of airway teachers in local departments

The main limitations for daily airway teaching and challenges perceived by TAT-course participants in their departments are lack of resources (e.g. dedicated time, equipment, personnel—63%), and lack of departmental support for teaching (54%; compare Fig. 1). Half of the survey participants rated their overall departmental level of preparation of airway teachers as good or excellent and 21% considered it inadequate (Fig. 2).

## Discussion

This survey revealed that based on participants’ self-assessment they changed their teaching approach in airway management after having participated in the TAT-course. This reflects a significant impact of the Teach-the-Airway-Teacher (TAT)-course of the European Airway Management Society (EAMS) on their airway teaching skills. Participants expressed a strong need for a more systematic approach to teaching airway management and reliable resources dedicated to airway management teaching in their institutions. These responses underline the effectiveness of the TAT-course as participants changed their teaching, but also provided feedback that enabled to improve the course. With that, we achieved the aims of our survey.

### TAT-participants’ characteristics and opinions

Because most of the TAT-course participants were already experienced teachers (mean 9 years) before participating in the TAT-course, we value the reported changes in the TAT-course participants approach to teaching after the course as an important sign of the specific impact of the TAT-course on their clinical airway teaching skills. As summarized in Table 3 it seems that classic didactic lectures are less useful. On the other hand, these results suggest favouring the teaching of non-technical skills and to provide effective feedback, probably because these topics are usually less covered in other courses.

In fact, TAT-course participants agree that modern airway management needs theoretical knowledge, regular training of technical skills with the daily used airway management devices, and constant practice and training of human factors, especially leadership and effective communication in order to successfully solve an airway crisis when it arises [20]. Clinicians have expressed the need to be educated in all these competencies [21].

Every specialist involved in airway management needs to stay competent in proper difficult airway management. As a result, trainees have fewer opportunities to learn and train to manage difficult airway situations during clinical rotations [22]. Therefore, it is important to teach trainees the necessary airway management skills in a dedicated educational setting to ensure their ability to safely manage a critical airway situation as responsible physicians, even if they have not encountered such clinical cases during their specialist training. While simulation is useful for training human factors, such as leadership and communications skills, airway management skills still need to be learned, trained and practiced on real patients with their naturally occurring differences in anatomy and physiology. Making safe decisions for difficult airway management requires experience.

The qualitative results of the open-ended questionnaire showed that participants mainly changed their approach to adult learning and how to improve their own teaching and feedback as well as debriefing. This again reflects the effectiveness of the TAT-course. Additionally, participants oriented themselves more toward the student and wanted to overcome barriers to teaching and address organisational barriers, which shows how the TAT-course impacts course participants on a personal level.

### Airway teaching in departments

On-site face-to-face learning was preferred by three-quarters of the survey participants (Table 4), and this is available in 87% of departments (Table 1). However, there is still a lack of structure in airway teaching at a departmental level, insufficient resources are dedicated to teaching airway management. Also, there is a lack of structured training for airway management teachers, as only 40% of clinical teachers have received specific education for teaching airway management (Table 4).

Even in departments where the teachers are adequately trained, there are still barriers to successful airway management teaching such as a lack of resources (e.g., space, equipment, dedicated time for teaching etc.). This is probably a reason for the low frequency of departmental courses reported: 1–2 times per year in 68% of departments,16% with more than 2 courses per year, and 16% that did not organize any courses.

Despite those deficiencies, departments, curricula and last but not least clinical teachers need to assure that learners in airway management are provided with sufficient opportunities to learn and train in core airway management skills, ideally in a structured and compulsory way [23]. Relying purely on the chance to encounter difficult airway situations during a residency or during clinical practice is an insufficient way to maintain airway management competencies. If this is done, there might be consequences for patient safety.

### Limitations

There are some limitations to our survey: The survey had a 56% response rate, and therefore, there is the potential for a selection bias as participants perceiving the course either as very helpful or as less helpful might have an interest in providing their feedback. One sign of a potential bias toward participants who found the course very helpful was that the percentage of certified airway teachers was higher in the study cohort compared to the overall number of certifications issued after the respective TAT-courses. Moreover, most TAT participants that completed the survey were anaesthetists, testifying to a possible need to get also intensive care and emergency physicians involved in the TAT-course, as difficult airways are likely to be encountered and managed in those settings as well. Another limitation is that we were only able to assess the airway teacher’s perspective, we were not able to obtain the opinion of the students of the airway teachers included in this survey. Therefore, we cannot comment if the quality of the airway management training really improved for the TAT-course participants’ learners and if the learners would agree that the subjectively perceived improvements have been implemented successfully.

## Conclusions

This survey confirms the impact of the Teach-the-Airway-Teacher (TAT)-course on translation and implementation of airway management teaching of clinical teachers in their home institutions. After the course participants substantially changed their way of teaching airway management. Structured airway management curricula and airway management teacher education was acknowledged as most important to improve patient care. Feedback obtained in the survey will improve future TAT-courses’ content and delivery.

### Supplementary Information

**Additional file 1.**

## Data Availability

The data can be made available upon reasonable request from the corresponding author following approval of the local ethical committee.

## Abbreviations

EAMS: European Airway Management Society
TAT: Teach-the-Airway-Teacher
