# Supplementary material for: Teaching airway teachers: a post-course quantitative and qualitative survey
Source: BMC Med Educ. 2024 Feb 7;24:123. doi: 10.1186/s12909-023-04912-y (PMC10848376; doi:10.1186/s12909-023-04912-y)
Supplement: Supplementary file 1 — Additional file 1. [file 12909_2023_4912_MOESM1_ESM.docx]

**Post-TAT Course Questionnaire**

**(Teach the Airway Teacher Course)**

**SECTION 1. PERSONAL AIRWAY TEACHER BACKGROUND**

1. How long have you been practicing as a physician after completing your training as a specialist?

o Please provide the number of years

2. In which setting do you practice most of your clinical activity (>50% of the time)? (please select one)

o Intensive Care Unit

o Anaesthesia

o Emergency Department

o Pediatrics

o Research

o Education

o Other (please specify)

3. How long have you been practicing as an airway teacher?

o Please provide the number of years

4. In which kind of hospital/department are you practicing?

o University-based hospital

o Non-University based hospital

o Office based practice

o Other (please specify)

5. Which students do you usually teach in airway management? (Multiple answers are possible)

o Medical students

o Residents

o Specialized physicians

o Paramedics

o Nurse students

o Certified nurses

o Others (please specify)

6. Which type of Teach the Airway Teacher (TAT) course did you attend?

o Face-to-face in presence TAT

o Hybrid blended TAT

o Both

**SECTION 2. AFTER TAT OVERVIEW**

7. When did you attend your Teach the Airway Teacher (TAT) course?

o Year:

8. Did you finish your post-course assignments, and did you obtain the EAMS Airway Teacher certificate?

o Yes

o No

9. How many airway courses have you been teaching after your TAT course?

o Please provide a number

10. Are you teaching structured airway management in clinical practice on regular bases?

o Never

o Daily

o Weekly

o Monthly

o 1-2 times/year

o Others (please specify)

11. Did you change your way of teaching after attending a TAT course?

o Not at all

o Yes, in some respects

o Yes, completely

12. If you answered yes to question 11: Which were the specific changes?

Open question

13. What are the main limitations or challenges in your airway teaching? (Multiple answers are possible)

o Interaction with the audience

o Effective presenting topics

o Keeping learners' attention to the point

o Setting a stimulating learning climate

o Proper assessment

o Giving effective feedback and debriefing

o Time management

o Institution (hospital) does not provide protected teaching time

o Missing equipment

o Missing teaching locations (rooms)

o Not enough participants

o Other (please specify)

14. What are your strengths as an airway teacher? (Multiple answers are possible)

o My continuous lifelong learning

o Knowledge of educational “theory” (adult learning, lesson structure, etc.)

o Empathy with students

o Effectiveness in presenting

o Engagement with learners

o My airway management skills

o My status as an airway expert

o Empathic feedback and debriefing

o Goal-oriented teaching

o Other (please specify)

15. How important do you consider continuous education (like the TAT courses) for your career?

o Unnecessary

o Sometimes useful

o Quite useful

o Crucial

o Do not know

16. Which educational topic do you consider most effective in the TAT course? (Multiple answers are possible)

o Learning about the role of the teacher

o “Educational Theory”

o The teaching of non-technical skills

o The teaching of airway management skills

o Lecturing

o Use of blended learning methods (mix of session formats)

o Micro-teaching exercise

o Feedback exercise

o How to run an airway course

o Other (please specify)

17. Which educational topic did you find less helpful for your airway teaching? (Multiple answers are possible)

o Learning about the role of the teacher

o “Educational Theory”

o The teaching of non-technical skills

o The teaching of airway management skills

o Lecturing

o Use of blended learning methods (mix of session formats)

o Micro-teaching exercise

o Feedback exercise

o How to run an airway course

o Other (please specify)

18. Considering your airway teaching experience, what do you think is missing from the TAT program?

o Open question

**SECTION 3. LOCAL TEACHING REALITIES OVERVIEW**

19. How often does your department or clinical unit organize airway management courses each year (excluding the COVID-19 pandemic period)?

o Never

o 1-2 times per year

o > 2 times per year

20. How are these courses organized?

o In presence, theory

o E-learning, theory

o In presence, hands-on skills

o E-Learning, skills

o Blended courses

o Other (please specify)

21. Does your department have a standardized clinical airway teaching rotation for residents?

o Yes

o No

22. Does your department have a clinical “refresher” rotation for specialists in airway management?

o Yes

o No

23. Do the teachers in your department have any teaching certificates in airway teaching (clinical or courses)?

o Yes

o No

24. If yes, specify

25. How do you rate the overall level of preparation on airway teaching (didactic and airway skills) at your departmental educational activities?

o Inadequate

o Sufficient

o Good

o Excellent

o Do not know

26. What is the main limitation in your daily airway teaching? (Multiple answers are possible)

o Inadequate/not clear teachers’ training (certification)

o Inadequate teachers' preparation and/or updated competences

o Inadequate settings (rooms, multimedia, etc.)

o Inadequate learners’ preparation or selection

o Insufficient departmental support for teaching

o Insufficient teaching resources (manikins, simulation, dedicated time and personnel, etc.)

o Other (please specify)

27. Which is your favorite teaching modality? (multiple answers are possible)

o E-learning, virtual teaching, apps, … (technology-enhanced learning)

o On-site face to face learning

o Blended hybrid methods

o Airway courses externally (conferences, seminars, …)

o Continuous airway programs in my unit

o Clinical rotations with special learning topics

o Dedicated airway fellowships

o Other (please specify)

28. In your opinion what is needed to implement a local successful airway-teaching program (courses, rotations, hands-on sessions, etc.)?

o Open question
